# Citation Advantage of Open Access Articles

**DOI:** 10.1371/journal.pbio.0040157

**Published:** 2006-05-16

**Authors:** Gunther Eysenbach

**Affiliations:** **1**Centre for Global eHealth Innovation, University Health Network; and Department of Health Policy, Management and Evaluation, University of Toronto, Toronto, Ontario, Canada; University of TennesseeUnited States

## Abstract

Open access (OA) to the research literature has the potential to accelerate recognition and dissemination of research findings, but its actual effects are controversial. This was a longitudinal bibliometric analysis of a cohort of OA and non-OA articles published between June 8, 2004, and December 20, 2004, in the same journal *(PNAS: Proceedings of the National Academy of Sciences).* Article characteristics were extracted, and citation data were compared between the two groups at three different points in time: at “quasi-baseline” (December 2004, 0–6 mo after publication), in April 2005 (4–10 mo after publication), and in October 2005 (10–16 mo after publication). Potentially confounding variables, including number of authors, authors' lifetime publication count and impact, submission track, country of corresponding author, funding organization, and discipline, were adjusted for in logistic and linear multiple regression models. A total of 1,492 original research articles were analyzed: 212 (14.2% of all articles) were OA articles paid by the author, and 1,280 (85.8%) were non-OA articles. In April 2005 (mean 206 d after publication), 627 (49.0%) of the non-OA articles versus 78 (36.8%) of the OA articles were not cited (relative risk = 1.3 [95% Confidence Interval: 1.1–1.6]; *p* = 0.001). 6 mo later (mean 288 d after publication), non-OA articles were still more likely to be uncited (non-OA: 172 [13.6%], OA: 11 [5.2%]; relative risk = 2.6 [1.4–4.7]; *p* < 0.001). The average number of citations of OA articles was higher compared to non-OA articles (April 2005: 1.5 [SD = 2.5] versus 1.2 [SD = 2.0]; Z = 3.123; *p* = 0.002; October 2005: 6.4 [SD = 10.4] versus 4.5 [SD = 4.9]; Z = 4.058; *p* < 0.001). In a logistic regression model, controlling for potential confounders, OA articles compared to non-OA articles remained twice as likely to be cited (odds ratio = 2.1 [1.5–2.9]) in the first 4–10 mo after publication (April 2005), with the odds ratio increasing to 2.9 (1.5–5.5) 10–16 mo after publication (October 2005). Articles published as an immediate OA article on the journal site have higher impact than self-archived or otherwise openly accessible OA articles. We found strong evidence that, even in a journal that is widely available in research libraries, OA articles are more immediately recognized and cited by peers than non-OA articles published in the same journal. OA is likely to benefit science by accelerating dissemination and uptake of research findings.

## Abstract 2

A longitudinal bibliometric analysis of citations to papers published in the *PNAS* between June 8, 2004 and December 20, 2004 reveals that the open-access articles were more immediately recognized and cited by peers.

## Introduction

Open access (OA) to the scientific literature means the removal of barriers (including price barriers) from accessing scholarly work. There are two parallel “roads” towards OA: OA journals and self-archiving [ 1, 2]. OA journals make published articles immediately freely available on their Web site, a model mostly funded by charges paid by the author (usually through a research grant). The alternative for a researcher is “self-archiving” (i.e., to publish in a traditional journal, where only subscribers have immediate access, but to make the article available on their personal and/or institutional Web sites [including so-called repositories or archives]), which is a practice allowed by many scholarly journals.

OA raises practical and policy questions for scholars, publishers, funders, and policymakers alike, including what the return on investment is when paying an article processing fee to publish in an OA journal, or whether investments into institutional repositories should be made and whether self-archiving should be made mandatory, as contemplated by some funders [ 3].

Among the arguments of OA proponents (and an expectation of scientists who publish OA articles) is that “open” work is more quickly recognized, as measured by citations. Critics of OA dispute this fact and argue that there is “no evidence that this will happen.” [ 4] Representatives of traditional publishers argue that the “established system of scientific/technical/medical publishing provides excellent levels of open access to scientists and the public alike,” implying that scientists have access to the literature anyway and that there would be little advantage to publish OA. [ 5]

In fact, the evidence on the “OA advantage” is controversial. Previous research has based claims of an OA citation advantage mainly on studies looking at the impact of self-archived articles or articles that are found online (“openly accessible,” which some have argued to be different from open access in the narrower sense [ 6]). Most studies show an association between being online and being cited more often [ 1, 7– 9], although another study in the field of pediatrics seemed to suggest the opposite [ 10].

All these previous studies are cross-sectional and are subject to numerous limitations.

The first problem is self-selection. As most of these previous studies broadly define OA as “being found freely available online,” [ 7, 9] alternative explanations for citation differences include that important (high-citation) articles are more likely to be posted online by authors or users as a *result* of the articles' importance; for example, because they are used for journal clubs [ 6] or coursework, or because authors post them on their homepages because they get so many requests from peers (Wren found that online accessible papers are clearly biased towards publications with “higher popular demand” [ 6]). In other words, one could argue that the articles are online *because* they are highly cited, rather than being highly cited because they are online. A mere association in a cross-sectional study tells us nothing about the direction of the relationship. Kurtz even argues that “the claims that the citation rate ratio of papers openly available on the internet versus those not available is caused by the increased readership of the open articles…(“OA advantage”) are somewhat overstated.” [ 11] Similarly, while the usual line of argument is that self-archiving leads to higher citations [ 8], alternative explanations include that top authors are more likely to be at top institutions that may be more likely to have an institutional repository, which smaller institutions do not have, or that authors selectively self-archive their best work as a “trophy.” [ 6] A recent analysis of articles published in four mathematics journals indicates that articles deposited in the arXiv ( http://arXiv.org) received more citations than nondeposited articles, but the authors do not attribute OA as the cause of more citations, but self-selection (quality differential) [ 12].

Secondly, especially in fields like physics, where pre- and post-publication on http://arXiv.org is quasi-standard, a relationship between self-archiving and higher citation may be due to other factors, such as earlier dissemination of results through preprints [ 11], a quality improvement through discussion of preprints [ 13, 14], or an “outsider” position of authors who do not self-archive.

Thirdly, previous studies reported crude, unadjusted rate ratios, where differences in author and article characteristics between OA and non-OA publications were not taken into account and corrected for. One could argue that the observed citation advantages of self-archived papers are a result of confounders; for example, publications with more authors are more likely to be self-archived (as it takes only one author to self-archive) and are also (independently from any OA effect) cited more often (e.g., through increased self-citations or because they might be of higher quality).

Limited or no evidence is available on the citation impact of articles originally published as OA that are not confounded by the various biases and additional advantages of self-archiving or “being online” that contribute to the previously observed OA effects. A “journal-level” analysis of journal impact factors concluded that OA journals are more often in the lower half of their subject category, although within the collection of OA titles, these journals ranked higher by immediacy index than by impact factor [ 15]. However, comparing the impact of OA journals against non-OA journals ignores differences in the journals' novelty, editorial policies, quality of peer review, and acceptance policies, which are strong confounders that are difficult to adjust for.

To answer the question of whether OA publications lead to a citation advantage I chose an article-level approach, comparing the bibliometric impact of a cohort of articles from the same journal *(Proceedings of the National Academy of Sciences [PNAS])* that offers both an OA and a non-OA publishing option, adjusted for different article and author characteristics.

## Results

### Article and Author Characteristics

A total of 1,492 original articles were included: 212 (14.2% of all articles) were published as immediate OA articles on the journal site, and 1280 (85.8%) as non-OA articles. On December 31, 2004, the articles in the cohort had been published (in most cases, electronically before print publication) within the last 194 d (mean, 101 d; SD = 57.5), with OA articles being on average younger (83.6 d; SD = 50.2) than non-OA articles (104.0 d; SD = 58.1) ( *p* < 0.001) since OA publishing became more popular over time. OA articles had a higher number of authors ( *p* = 0.002) and were more likely to be track I or III than non-OA articles ( *p* = 0.002). There were no significant differences in terms of the granting organization ( *p* = 0.46) ( Table 1).

**Table 1:**
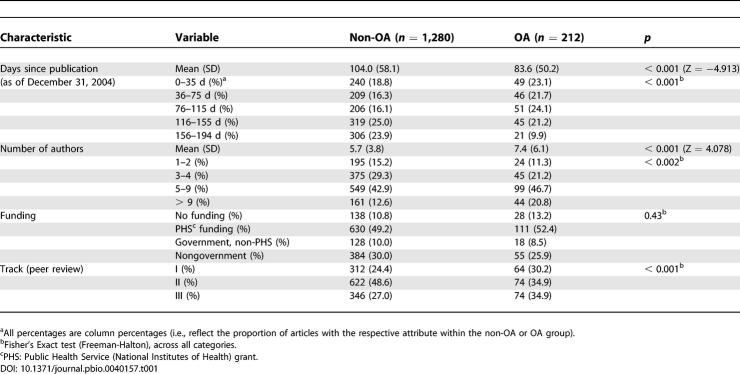
Article Characteristics

Authors came from 39 different countries, with the majority ( *n* = 981; 65.8%) from the United States. Together, 12 countries constituted 95% of all the articles, and all had a minimum of ten articles each; other countries were aggregated in an “other” category. Among these 12 countries, Japan had the highest proportion of OA articles (13/67; 19.4%), followed by Israel (3/16; 18.8%), Germany (11/70; 15.7%), and the United States (152/981; 15.5%). Countries with the lowest proportion of OA articles included Switzerland (0/22), the Netherlands (0/17), Sweden (1/31; 3.2%), the United Kingdom (7/84; 8.3%), and Canada (3/31; 9.7%).

The majority (1,304/1,492, or 87.4%) of papers were from one of 14 major subject areas; the remaining smaller subjects were aggregated into an “other” category. Biochemistry was the largest article category ( *n* = 190; 12.7%), followed by medical sciences ( *n* = 152; 10.2%) and neuroscience ( *n* = 149; 10.0%). The subject areas with the largest proportion of OA articles were microbiology (21/91; 23.1%), genetics (18/90; 20.0%), and evolution (12/71; 16.9%), while plant biology (2/53; 3.8%), chemistry (4/62; 6.5%), and biophysics (11/136; 8.1%) were subject areas with the lowest proportion of OA articles.

There was a borderline-significant trend towards OA first authors having more lifetime publications, with no significant differences between the groups for last authors' publication counts (first authors: OA median = 70.5 versus non-OA = 38.0; Z = 2.013, *p* = 0.0441; last authors: OA = 194.5 versus non-OA = 170.5; Z = 0.670, *p* = 0.503), perhaps pointing to the fact that first authors tended to be more senior in the OA group. There were significant differences between the groups in the authors' lifetime average citations per paper, with first authors being “stronger” in the non-OA group, and last authors being better in the OA group (first authors: OA median = 7.77 versus non-OA = 8.98; Z = 2.304, *p* = .02; last authors: OA = 13.64 versus non-OA = 16.35; Z = 3.456, *p* < 0.001). An aggregate variable, indicating the average citation frequency of a paper from the first and last author combined, shows a borderline significant trend towards OA authors being cited more often per paper (OA = 12.31 versus non-OA = 10.02; Z = 2.001, *p* = 0.045). All variables were included in the multivariate models to adjust for these differences.

Among the 237 participants of the author survey (response rate, 75.4%), there were no statistically significant differences between the groups in self-rated relative urgency, importance, and quality of their particular *PNAS* article ( *p* > 0.05).

### Citations

#### Crude analysis.

In the crude analysis, the mean number of citations as well as the proportion of articles cited at least once was significantly higher in the OA group in both the April 2005 and the October 2005 analyses, with the relative “risk” for non-OA articles of not being cited increasing over time ( Table 2).

**Table 2:**
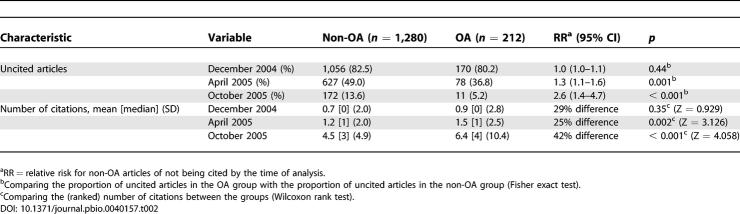
Crude (Unadjusted) Analysis

In an analysis stratified by subject, there was a trend for an OA citation advantage in almost all subjects, although, due to the limited number of articles per subject area, few of these differences reached statistical significance (unpublished data).

#### Adjusted analyses.

A number of potential confounders must be considered and adjusted for to correct for differences in the number of authors, past productivity (or author seniority), time since publication, and submission track, which differ between the article groups and could be independently related to the probability of getting cited.

As shown above, in the crude analysis, the proportion of uncited articles differed significantly between the groups at all three analysis points ( Table 2). In order to determine whether these differences remained significant when adjusted for potential confounders, a logistic regression predicting “cited” status as dependent variable and with stepwise backwards elimination of potential predictors and confounders that were not statistically significant was conducted, controlling for first and last author's lifetime publication count, first and last author's lifetime average citations per paper **,** number of days since publication (categorized), number of authors (categorized), country of the corresponding author (12 most common countries and “other”), funding type, subject area (14 most common subjects and “other”), and submission track. OA status remained an independent predictor for being cited for all three analysis points, with an increasing odds ratio over time in favor of OA articles ( Table 3).

**Table 3:**
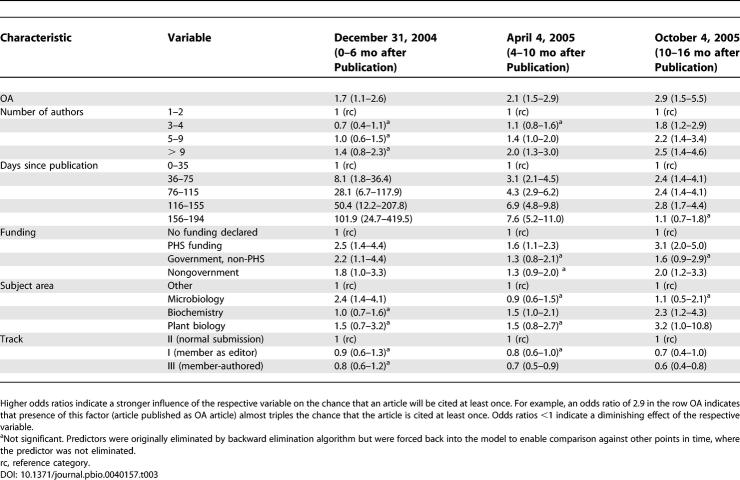
Odds Ratios (with 95% Confidence Intervals) of Significant Predictors from a Stepwise Backward Logistic Regression Model (for Three Points in Time) with “Cited” Status as the Dependent Variable and OA Status as Well as Potential Confounders as Independent Variables

Similarly, using a stepwise backward linear regression model including the same covariates, OA status remained a significant independent predictor for the number of citations (transformed on a logarithmic scale) both in the April 2005 analysis (beta for OA status = 0.187; *p* < 0.001; overall model fit *r ^2^* = 0.21) as well as in the October 2005 analysis (beta for OA status = 0.263; *p* < 0.001; overall model fit *r ^2^* = 0.15).

### Secondary Analysis

*PNAS* allows authors to “self-archive” their research on the Internet even if they choose the non-OA option. This means that some of the articles in the non-OA group may in fact have been “openly accessible” online through the author's homepage or an institutional repository. In a secondary analysis I also analyzed citations of “self-archived OA” articles (i.e., self-archived or otherwise openly accessible on other Web sites than http://www.pnas.org or http://pubmedcentral.org), with the explicit caveat that articles which are found on the Internet are subject to self-selection and other biases as discussed in the introduction (i.e., it is impossible to discriminate whether they are on the Internet because they are important, or whether they are highly cited because they are on the Internet).

Citation rates (as of October 2005) of four separate subgroups were analyzed, as an article could be either published under the *PNAS* immediate OA option or “self-archived,” or both, or none. There was a clear relationship between the level of openness and the citation levels ( Table 4).

**Table 4:**
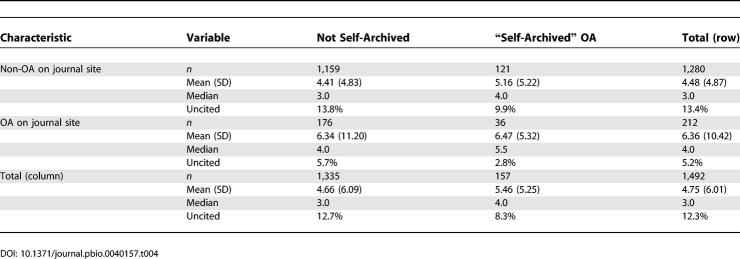
Secondary Analysis with “Self-Archived” (Openly Accessible Articles Found on the Authors' Homepages, in Institutional Repositories, or Elsewhere on the Internet) as Separate Subgroup (October 2005 Analysis, 10–16 mo after Publication)

While 36 of 212 (17.0%) of immediate journal OA articles were also self-archived, only 121 of 1,280 (10.6%) of non-OA papers on the journal site were self-archived (i.e., papers published originally as OA were more likely to be self-archived [Fisher's exact test *p* = 0.002]).

The 1,159 papers which were neither self-archived nor immediate journal OA articles had on average 4.4 citations, whereas the 334 papers which were either self-archived or published originally as OA (or both) had 5.9 (Z = 4.215, *p* < 0.001) citations. The risk of not being cited for papers which were either published originally as OA or self-archived was only 6.9%, while it was 13.81% for articles in the non-OA group (neither self-archived nor published originally as OA; relative risk = 2.0 [1.31–3.04]; Fisher's exact test *p* < 0.001).

While in the crude analysis self-archived papers had on average significantly more citations than non–self-archived papers (mean, 5.46 versus 4.66; Wilcoxon Z = 2.417; *p* = 0.02), these differences disappeared when stratified for journal OA status ( *p* = 0.10 in the group of articles published originally as non-OA articles, and *p* = 0.25 in the group of articles published originally as OA).

In a logistic regression model with backward elimination, which included original OA status and self-archiving OA status as separate independent variables as well as all potential confounders, self-archiving OA status did not remain a significant predictor for being cited. In a linear regression model, the influence of the covariate “article published originally as OA, without being self-archived” (beta = 0.250, *p* < 0.001) on citations remained stronger than self-archiving status (beta = 0.152, *p* = 0.02).

## Discussion

### Main Findings

This comparison of the impact of OA and non-OA articles from the same journal in the first 4–16 mo after publication shows that OA articles are cited earlier and are, on average, cited more often than non-OA articles. To my knowledge, this is the first longitudinal study of a cohort of OA and non-OA articles providing direct and strong evidence for preferential or earlier citation of articles published originally as OA. It is also the first study showing an advantage of publishing an article as OA on the journal site over self-archiving (i.e., making the article otherwise online accessible).

The strength of the OA effect is particularly surprising because *PNAS* is a widely available journal that is accessible for most researchers through their library. In addition, articles are made freely available to nonsubscribers 6 mo after publication. The effect of OA publishing may be even higher in fields where journals are not widely available and where articles from the control group remain “toll-access.”

### Limitations

This study offers only a short-term glimpse at what happens at the left-hand (early) side of the temporal citation curve. A citation curve plotting over time the number of new citations per year to articles published in a certain year would show a sharp increase of new citations with a citation peak after 1 or 2 y (which is typically the time needed for citing authors to prepare and publish their papers), with a slow and steady decline of new citations year after year. Despite the narrow observation window of this study, it appears that publishing OA does not merely lead to a steeper increase and “left-shift” of the citation curve by 6 mo, because such a left-shift is incompatible with the observation in this study that there are still increased citation rates for OA articles at our third observation point 10–16 mo after publication. Rather, there seems to be a sustained effect on the absolute number of citations. In other words, there seems to be not only an advantage in terms of immediacy (defined as the average number of times that an article, published in a specific year within a specific journal, is cited over the course of the same year), but also in terms of total impact (as measured by the absolute number of citations received over a longer period of time). Future follow-up analyses following this cohort over a number of years will provide a more complete picture on how long-lasting the citation advantage of OA articles is.

As this was an observational study and not a randomized trial, we were able to statistically control only for known confounders. There is a possibility of selection bias in authors judging the importance of their work and making a deliberate decision to publish their most important work as OA, with quality differences between articles contributing to citation differences. However, results from our author survey—in which we asked authors to self-rate the importance and quality of their work—did not show significant differences between the groups and do not make this likely, in particular because *PNAS* is a high-impact journal and most of the authors considered their work high quality in the first place.

*PNAS* has a broad interdisciplinary scope and different submission tracks, which are features that should enhance the generalizability of the results. However, it has to be acknowledged that our data come from a single, rather atypical journal, and should be replicated with data from other newer hybrid (“author-choice OA”) journals. Other publishers have started to offer an author-choice option, but only recently and with limited sample sizes both in terms of articles and in terms of citations. While a study on these journals is under way, these results will be available only in a few years.

This study used citations as a proxy for impact, and some may argue that “it is hard to see how science will benefit by increased citation rates [of OA articles].” [ 4] Our data do suggest that OA articles are more quickly recognized and their results are picked up and discussed by peers to a larger extent. It is hard to see how faster and increased utilization and uptake of research results will *not* benefit science, at least in terms of accelerating the speed by which new results are verified, falsified, or built upon by others. By focusing on citations this study only addresses the impact on other research users, not on the knowledge user (i.e., policymakers, consumers, or health professionals), but it can be hypothesized (and should be tested in future studies) that there is also a “knowledge translation advantage” in terms of increased and accelerated knowledge uptake by consumers and policymakers.

### Conclusions

OA journals and hybrid journals like *PNAS,* as well as traditional publishers like Blackwell Publishing (“Online Open”), Oxford University Press (“Oxford Open”), and Springer (“Springer Open Choice”) are now offering authors an immediate OA option if the author pays a fee. Researchers, publishers, and policymakers confronted with the question of whether or not to invest in OA publishing have reason to believe that OA accelerates scientific advancement and knowledge translation of research into practice. While more work remains to be done to evaluate citation patterns over longer periods of time and in different fields and journals, this study provides evidence and new arguments for scientists and granting agencies to invest money into article processing fees to cover the costs of OA publishing. It also provides an incentive for publishers seeking to increase their impact factor to offer an OA option.

The findings indirectly also support policies of granting agencies which made (or consider to make) OA publishing (be it only through self-archiving) mandatory for grantees [ 3], as it illustrates the advantage of openess in the dissemination of knowledge. However, this study suggests that publishing papers as OA articles on the journal site facilitates knowledge dissemination to a greater degree than self-archiving, presumably because few scientists search the Internet or Google for articles if they have encountered an access problem on the journal Web site.

## Materials and Methods

### Article cohort.

*PNAS* announced on June 8, 2004, that authors could pay US$1,000 if they wanted their article to be immediately OA [ 16] (as opposed to the usual non-OA “moving wall” model, where articles become freely accessible after 6 mo). The resulting mix of OA and non-OA articles published between June 8, 2004 (page 8745 of Volume 101), and December 20, 2004, in *PNAS* constituted the article cohort. Included were the article types “Journal Article,” “Clinical Trials,” and “Case Reports,” excluding editorials, commentaries, biographies, retractions, and retracted articles, “classics” papers, and supplement articles. To determine citation rates, Web of Science (Thomson ISI, Stamford, Connecticut) was searched on December 31, 2004, April 4, 2005, and again on October 4, 2005, for all articles citing papers from the cohort. Citation errors (e.g., wrong volume or misspelling of the author name) were manually corrected. The following article characteristics were extracted and used in the multivariate models in order to control for potential confounders: days since publication, number of authors, article type, country of the corresponding author, funding (as indexed in Medline), subject area (as classified in the *PNAS* Table of Contents), and submission track. *PNAS* has three different submission tracks: the majority (80%) of submissions are made through a regular submission track (called “track II”), where authors submit manuscripts to the editorial office, which assigns an Academy member as editor to guide the paper through peer review [ 17]. *PNAS* has two further unique peer-review and submission models, called track I (authors submit their work directly to an Academy member, who solicits two reviews and then sends it to the editorial office for publication) and track III (Academy members can send their own articles to the editorial office, accompanied by peer-review reports which they themselves solicited) [ 18]. Including the submission track into the multivariate models allows control of different levels of rigor in peer review and quality of the contributions.

To control for possible differences in “author quality,” information on the first and last authors' total number of published articles and lifetime citations up to 2004 was gathered from Web of Science and was also included as adjustment variables in the multivariate models.

In addition, in order to test equivalence of article quality between the OA and non-OA groups, all authors with published and valid e-mail addresses ( *n* = 313) received a Web-based survey in which they were asked to rate the relative urgency, importance, and quality of their particular *PNAS* article relative to other articles on a five-point Likert scale.

### Secondary analysis.

To identify “self-archived” articles I used a computer program (in-house development, see Acknowledgments) that conducted for each article a Google ( http://www.google.com) phrase search, with the first sentence from the results section of the article or, if this did not lead to any hits, the digital object identifier as query. An article was considered “self-archived” if the article was found on Web sites other than on http://www.pnas.org or http://pubmedcentral.org. While this is not a perfect method (as Web coverage by search engines is incomplete [ 19]), it is currently the best we have, and if an article is not accessible through Google, it may not be found by the research community anyway and may not be considered “openly accessible.”

### Statistics.

For crude comparisons of continuous variables (number of days since publication, number of authors, and citations), the nonparametric two-sided Wilcoxon Mann-Whitney test was used, as these data were not normally distributed. Proportions were compared using the Fisher's exact test, while categorical variables with multiple categories were compared between the two groups using the Freeman-Halton test (an extension of Fisher's exact test for r by c tables).

In multivariate models I tried to predict the number of citations from several article characteristics, including OA status, adjusted for potential confounders as independent variables. In a linear regression model, the number of citations as the dependent variable was transformed into a log scale, as the distribution was skewed, predicting log(citations+1). In a logistic regression model, the number of citations as the dependent variable was dichotomized into 0 (uncited) and ≥ 1 (cited).

All data were analyzed using SAS, version 8.02 (Cary, NC).

## Abbreviations

OA: open access
PNAS: *Proceedings of the National Academy of Sciences*

## References

[pbio-0040157-b001] Harnad S, Brody T, Vallieres F, Carr L, Hitchcock S (2004). The access/impact problem and the green and gold roads to open access. Serials Review.

[pbio-0040157-b002] Anonymous (2002 February). Budapest Open Access Initiative.

[pbio-0040157-b003] Anonymous (2005). RCUK position statement on access to research outputs.

[pbio-0040157-b004] Aronson JK (2005). Open access publishing: too much oxygen?. BMJ.

[pbio-0040157-b005] Hunter K (2005). Critical issues in the development of STM journal publishing. Learned Publishing.

[pbio-0040157-b006] Wren JD (2005). Open access and openly accessible: A study of scientific publications shared via the internet. BMJ.

[pbio-0040157-b007] Lawrence S (2001). Free online availability substantially increases a paper's impact. Nature.

[pbio-0040157-b008] Harnad S, Brody T (2004). Comparing the impact of open access (OA) vs. non-OA articles in the same journals. D-Lib Magazine.

[pbio-0040157-b009] Antelman K (2004). Do open access articles have a greater research impact?. College and Research Libraries.

[pbio-0040157-b010] Anderson K, Sack J, Krauss L, O'Keefe L (2001). J Electr Publ.

[pbio-0040157-b011] Kurtz MJ, Eichhorn G, Accomazzi A, Grant C, Demleitner M (2005). The effect of use and access on citations.

[pbio-0040157-b012] Davis PM, Fromerth MJ (2006). Does the arXiv lead to higher citations and reduced publisher downloads for mathematics articles? [draft manuscript. Last updated Mar 20, 2006].

[pbio-0040157-b013] Eysenbach G (2000). The impact of preprint servers and electronic publishing on biomedical research. Curr Opin Immunol.

[pbio-0040157-b014] Eysenbach G (1999). Challenges and changing roles for medical journals in the cyberspace age: Electronic pre-prints and e-papers. J Med Internet Res.

[pbio-0040157-b015] McVeigh ME (2004). Open access journals in the ISI citation databases: Analysis of impact factors and citation patterns.

[pbio-0040157-b016] Cozzarelli NR (2004). An open access option for PNAS [editorial]. Proc Natl Acad Sci U S A.

[pbio-0040157-b017] Cozzarelli NR (2003). PNAS at volume 100 [editorial]. Proc Natl Acad Sci U S A.

[pbio-0040157-b018] Anonymous (2006). PNAS Information for Authors.

[pbio-0040157-b019] Lawrence S, Giles CL (1999). Accessibility of information on the web. Nature.

[pbio-0040157-b020] Eysenbach G, Trudel M (2005). Going, going, still there: Using the WebCite service to permanently archive cited web pages. J Med Internet Res.

